# 3D printed optimized electrodes for electrochemical flow reactors

**DOI:** 10.1038/s41598-024-71765-w

**Published:** 2024-09-30

**Authors:** Jonathan T. Davis, Buddhinie S. Jayathilake, Swetha Chandrasekaran, Jonathan J. Wong, Joshua R. Deotte, Sarah E. Baker, Victor A. Beck, Eric B. Duoss, Marcus A. Worsley, Tiras Y. Lin

**Affiliations:** Lawrence Livermore National Laboratory, Livermore, CA 94550 USA

**Keywords:** Optimized electrodes, Inverse design, Electrochemical reactors, Flow batteries, 3D Printing, Porous electrodes, Electrochemistry, Energy storage, Chemical engineering, Porous materials, Electrochemistry

## Abstract

Recent advances in 3D printing have enabled the manufacture of porous electrodes which cannot be machined using traditional methods. With micron-scale precision, the pore structure of an electrode can now be designed for optimal energy efficiency, and a 3D printed electrode is not limited to a single uniform porosity. As these electrodes scale in size, however, the total number of possible pore designs can be intractable; choosing an appropriate pore distribution manually can be a complex task. To address this challenge, we adopt an inverse design approach. Using physics-based models, the electrode structure is optimized to minimize power losses in a flow reactor. The computer-generated structure is then printed and benchmarked against homogeneous porosity electrodes. We show how an optimized electrode decreases the power requirements by 16% compared to the best-case homogeneous porosity. Future work could apply this approach to flow batteries, electrolyzers, and fuel cells to accelerate their design and implementation.

## Introduction

Electrification is necessary for decarbonizing many industrial chemical processes, but the design rules to achieve this are not always clear^1^. In electrochemical devices, high-performing electrode designs are often optimized for a specific material property—such as kinetic rate constant^2^, mass transfer coefficient^3^, or charge conductivity^4^. A better design approach for electrochemical reactors would also account for bulk material concentrations, electrical energy requirements, fluid pumping requirements, and integration with the external manifolding^5^. However, the relationship between basic material properties and these global metrics can be complex, and thus, successfully integrating the physics of each of these processes to yield an optimal design can be tedious^6^. This is especially true for porous electrodes, where the performance is known to be strongly affected by its 3D architecture across the nano-, micro- and meso- length scales^7^. Properly defining these relationships can be obscured by a lack of control over the electrode structure itself, which is often disordered. It is therefore important to develop new tools that can guide the design of porous electrodes and understand how the underlying micro-architecture can impact the overall device performance.

Advanced manufacturing has emerged as a tool to develop materials with spatially-varying porosities at increasingly fine length scales^8–10^. Alongside these advances, inverse design models have been developed to optimize shapes and pore structures based on simulations of relatively simple physical phenomena^11–13^. This is in contrast to conventional design approaches, where an initial structure is proposed and then incrementally improved upon through experimental trial and error. Inverse design establishes an objective function first and then solves for an optimal structure to maximize performance. This approach has been demonstrated for lightweighting^14^, heat sinks^15,16^, and optical devices^17,18^. Theoretical models have also been proposed for inverse design of components for electrochemical flow systems^19–22^, but these efforts have not yet been experimentally realized.

Experimental validation of an optimized flow electrode is difficult in part due to the coarse feature size resolution of electrically conductive 3D printed parts. However, significant materials science advances in this space have shown promises of improved resolution and design relevance of functional 3D electrodes^23^. Functional 3D printed electrodes have been demonstrated for use in flow batteries^3,24–26^, water electrolyzers^27–29^, fuel cells^30^, and supercapacitors^31,32^. Some of the most detailed functional structures to be printed are glassy carbon lattices^33^. A modified procedure by Kudo et al. has shown that it is possible to manufacture carbon lattices using projection microstereolithography (PuSL) coupled with pyrolysis, and their mechanical testing showed that these lattices are strong: uniaxial compression tests showed a strength of 152 MPa^34^. These structures have been shown to be electrochemically active^30,35–37^. Most of this research remains in the proof-of-concept phase, but using optimization tools can help identify carbon lattice structures which give superior performance. Applying this approach to scaled-up systems would allow engineers to rapidly identify the most important design features, even under mixed control regimes with no clear single driver of efficiency. These features can be adapted to more scalable manufacturing methods—CNC milling, injection molding, stacking of simpler geometries, etc.—to make analogous structures which capture the energy savings of an optimized design. Additional research is necessary to understand how transitioning to mass manufacturing approaches affects the final performance of the device.

This manuscript presents the first experimental demonstration of 3D printed flow-through electrodes with a pore structure and associated performance improvement predicted by inverse design. Recently, Reale Batista et al. demonstrated how 3D printed scaffolds can improve the active surface area for super capacitors in comparison to a periodic structure^38^. However, there has not yet been experimental validation for computationally optimized 3D electrodes against simulated data^5^. We establish this proof in practice for flow-through electrodes carrying out a continuous electrochemical reaction. The outcome of this approach is the ability to design and manufacture 3D electrodes with heterogeneous porosity which can outperform electrodes with a homogeneous porosity. Validation is demonstrated with 3D printed carbon lattice electrodes that are continuously reducing ferricyanide. As a model single phase reaction, experiments on known pore structures can be readily compared against continuum scale electrochemical models.

We further describe how the electrodes are designed, manufactured, and evaluated. First, we go over the design framework, providing the theoretical basis of the optimization for power loss minimization. The physical dependencies on porosity are described. Next, we describe the manufacturing process, detailing how functional electrodes are made. Lastly, we evaluate the electrochemical performance of the electrodes in a flow cell. We explore the tradeoff between electrical power and flow power losses in response to changes in operating parameters.

### Computational optimization framework

**Fig. 1:**
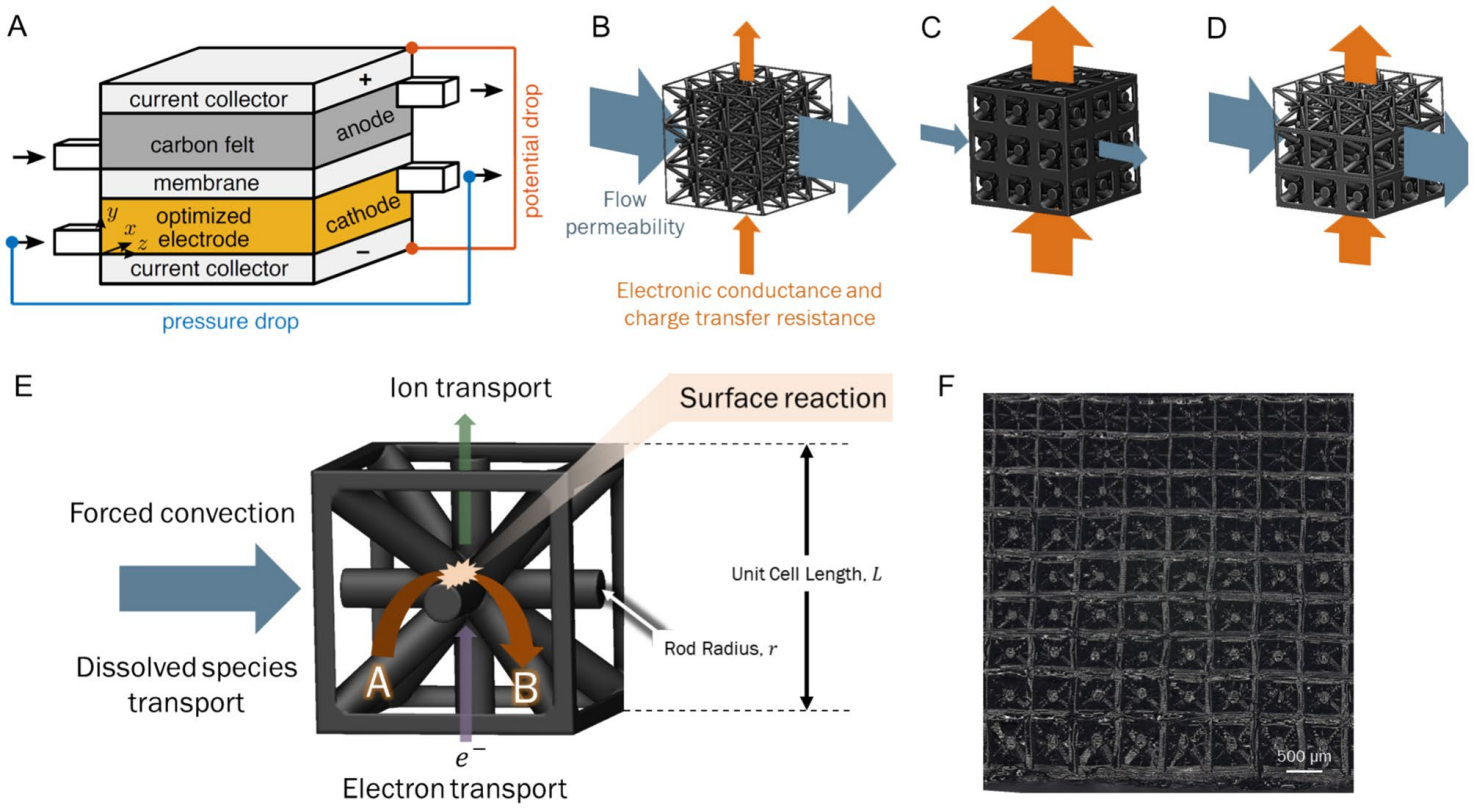
(**A**) Schematic of the electrochemical flow cell. The porosity of the 3D printed lattice cathode is optimized to reduce the power losses from the pressure and potential drops. Porosity control is achieved through tuning the thickness of the constituent rods within a lattice. (**B**) Schematic of an electrode with thin rods. Arrows indicate high permeability to liquid flow but low electronic conductivity and reaction surface area. (**C**) Schematic of an electrode with thick rods, which has a low permeability to fluid flow but offers higher electronic conductivity and reaction surface area. (**D**) Schematic of an electrode with a heterogeneous distribution of rod sizes which can achieve an optimal balance fluid permeability, charge conductivity, and reaction surface area. (**E**) Schematic of the transport processes taking place within an individual unit cell of a 3D printed electrode. These processes are simulated using continuum scale modeling. (**F**) Image of a 3D printed electrode with a gradient of rod thicknesses.

We consider the electrochemical flow cell shown schematically in Fig. 1A, and we focus on optimizing the cathode electrode to minimize total power losses. Within this domain, changes in porosity influence the power losses associated with fluid transport and electrical processes. A high porosity lattice, shown in Fig. 1B, is more permeable to fluid flow but is also more resistive to electronic transport. Conversely, a lower porosity lattice, shown in Fig. 1C, is less permeable to fluid flow but is more electrically conductive and can have a higher surface area for the electrochemical reaction. An optimal balance between the flow and electrical power losses can be achieved by creating electrodes with heterogeneous porosities (Fig. 1D). This work determines the optimal porosity distribution of the electrode by using continuum scale models to simulate the governing physical processes.

A summary of these physical processes is shown in Fig. 1E. Simulations of the electrode incorporate fluid flow, mass transfer, and electrostatics in tandem with an optimization of the spatial distribution of the lattice rod radii, i.e. the local porosity. Next, we fabricate the optimized design using 3D printing and image the electrode to ensure that the optimized porosity field was indeed experimentally realized. An example of a 3D printed electrode with a gradient in beam thicknesses is shown in Fig. 1F. Finally, we conduct electrochemical testing of the electrode. For simplicity, we focus our analysis on the optimization of the cathode half cell, following the work from Beck^5^. Experimentally, the performance of the cathode electrode was isolated from the anode and membrane by using a reference electrode. Although this study limits its scope to the optimization of a single electrode, future work could explore the co-optimization of both electrodes and their integration with surrounding components, including membranes, flow fields, and back contacts.

In the remainder of this section, we discuss the modeling and optimization approach. Liquid electrolyte, comprised of a solution of ferricyanide and ferrocyanide both at a concentration of ${C}_{inlet}=1 \text{ mM}$ flows into the cathode at a flow rate $Q$, where the reaction ${\left[Fe{\left(CN\right)}_{6}\right]}^{3-}+{e}^{-}\leftrightarrow {\left[Fe{\left(CN\right)}_{6}\right]}^{4-}$ occurs on the solid surfaces of the porous electrode. The reacted fluid then flows from the outlet. The modeling and optimization methodology closely follows the procedure outlined in Beck et al.^5^, which is summarized here briefly.

We aim to design the spatial distribution of rod diameters to minimize the total power loss $P$ for a given flow rate $Q$ and specified current density $I/A$. The power loss is defined to be

$$P={\int }_{\text{membrane}}\eta \frac{I}{A}d{\varvec{x}}+{\int }_{\text{inlet}}p\overline{u}d{\varvec{x} },$$

where $\eta$ is the cathode half-cell overpotential, $A=4 \text{ cm}^{2}$ is the membrane area, $p$ is the fluid pressure at the inlet, and $\overline{u }=Q/{A}_{\text{inlet}}$ is the average inlet velocity. The first integral refers to the electrical power losses, and the second integral refers to the power losses due to fluid pumping. Generally, an electrode with higher porosity will reduce the fluid pumping losses, but increase the electrical power losses, whereas an electrode with lower porosity will experience the opposite. In this work, we explore how an electrode with a spatially-varying porosity can aim to reduce the sum of the two.

Following Beck et al. (2021)^5^, we consider a porous electrode comprised a lattice of isotruss unit cells. Each unit cell has a side length $L$ and is comprised of rods of radius $r({\varvec{x}})$. While the side length is the same for each unit cell, the rod radius is allowed to vary across unit cells between ${r}_{min}$ and ${r}_{max}$. In this work, $L=690\, \mu \text{m}$, ${r}_{min}=22 \,\mu \text{m}$, and ${r}_{max}=102 \, \mu \text{m}$, and we consider ${N}_{x}\times {N}_{y}\times {N}_{z}=29\times 29\times 7$ total unit cells. The ratio $r/L$ thus controls the local porosity $\epsilon ({\varvec{x}})$ of the electrode, and we seek the distribution of porosity within the electrode to minimize $P$. In other words, we solve

$$\underset{{r}_{\mathit{min}}\le r\left({\varvec{x}}\right)\le {r}_{\mathit{max}}}{\text{min}}P,$$

$$\text{s}.\text{t}. {{\varvec{R}}}_{\left\{{\varvec{u}},p,{\phi }_{1},{\phi }_{2}{C}_{i}\right\}}=0,$$

where the ${{\varvec{R}}}_{\left\{{\varvec{u}},p,{\phi }_{1},{\phi }_{2}{C}_{i}\right\}}=0$ represents the forward problem that governs the physics. Specifically, we follow porous electrode theory^39^ and solve the homogenized governing equations: the incompressible Navier-Stokes equation modified with a Darcy drag for the fluid velocity ${\varvec{u}}$ and pressure $p$, the Poisson equations for the solid and liquid potentials ${\phi }_{1}$ and ${\phi }_{2}$, and an advection-diffusion-reaction equation for the oxidant and reductant concentrations ${C}_{i}$. The model describing the forward problem is summarized in the Methods and is implemented in OpenFOAM. A summary of the parameters used for simulating and optimizing the porous cathode is provided in Table 1 below. Notably, the local porosity $\epsilon ({\varvec{x}})$ modifies the local liquid and solid conductivities, the local mass diffusivities, the local permeability, and the local mass transfer coefficient from the liquid to the solid surfaces. The mass transfer coefficient is dependent on the interstitial velocity and flow profile within each unit cell, and this relationship was originally simulated by Beck^5^. To solve the optimization problem, the continuous adjoint approach is used. The adjoint problem corresponding to each forward equation is derived analytically, and these are used to calculate the sensitivities $dP/dr$. The Method of Moving Asymptotes^40^ is then used to iteratively update the electrode porosity field. A typical optimization simulation required approximately 6 h on 72 compute cores.

**Table 1:** Physical parameters for computational model.

Symbol	Parameter	Value	Units	Ref
$r$	Rod radius	22–102	µm	Figure 3
$L$	Unit cell length	690	µm	Figure 3
${\sigma }_{0}$	Electrode conductivity	4713	S/m	Figure S2
${\kappa }_{0}$	Electrolyte conductivity	10.9	S/m	^41^
${i}_{0}$	Exchange current density	1.93	A m^−2^	^41^
$\rho$	Electrolyte density	1000	kg m^−3^	Assumed
$\mu$	Electrolyte viscosity	8.91 × 10^−4^	Pa s	Assumed
${C}_{R}$	Ferricyanide inlet concentration	1	mM	Assumed
${C}_{O}$	Ferrocyanide inlet concentration	1	mM	Assumed
${D}_{R}$	Ferricyanide diffusivity	7.26 × 10^−10^	m^2^s^−1^	^41^
${D}_{O}$	Ferrocyanide diffusivity	6.77 × 10^−10^	m^2^s^−1^	^41^
$T$	Temperature	300	K	Assumed

**Fig. 2:**
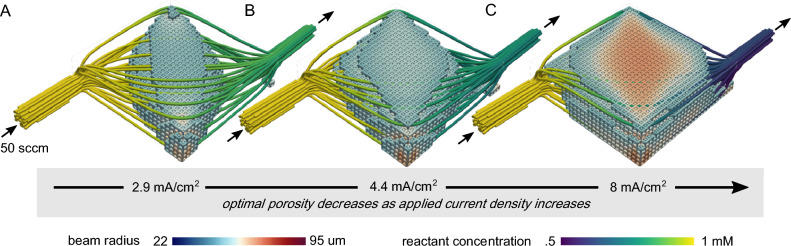
Electrodes optimized for a flow rate of $Q=50 \text{ sccm}$ for a current density of (**A**) $I/A=2.9 \text{ mA}/{\text{cm}}^{2}$, (**B**) 4.4 $\text{mA}/{\text{cm}}^{2}$, and (**C**) 8 $\text{mA}/{\text{cm}}^{2}$. To aid in visualization, only the portion of the electrode where the beam radius is $r>50 \, \mu \text{m}$ is shown. Additionally, streamlines of the flow, colored by the reactant concentration are shown.

The resulting porosity field from this approach is both a function of the material properties of the system and the choice of operating conditions. Figure 2 shows a series of optimized electrode designs corresponding to different operating current densities. As the operating current density increases, the average optimized beam thickness of the electrode increases. This is a result of the growing importance of electrical power losses, which the optimization algorithm compensates for by increasing the beam thickness, in order to both increase the effective electrode conductivity and specific surface area. However, the flow field lines also show a decrease in reactant concentration across the electrode, underscoring the importance of electrode structure for distributing mass and flow.

The flow field lines also show how forced convection laterally distributes reactants across the width of the electrode. For the cases shown in Fig. 2, the optimized porosity distribution is clearly a response to the positioning of the inlet and outlet and their associated entrance/exit region effects. In larger scale flow reactors, integrating components such as flow distributors or ribbed flow field plates will likely lead to different optimal porosity distributions. Nonetheless, the optimal porosity must still account for the concentration gradients, velocity profile, and mass transfer coefficients in order to make effective use of the entire electrode area.

### Manufacturing of heterogeneous porosity electrodes

**Fig. 3:**
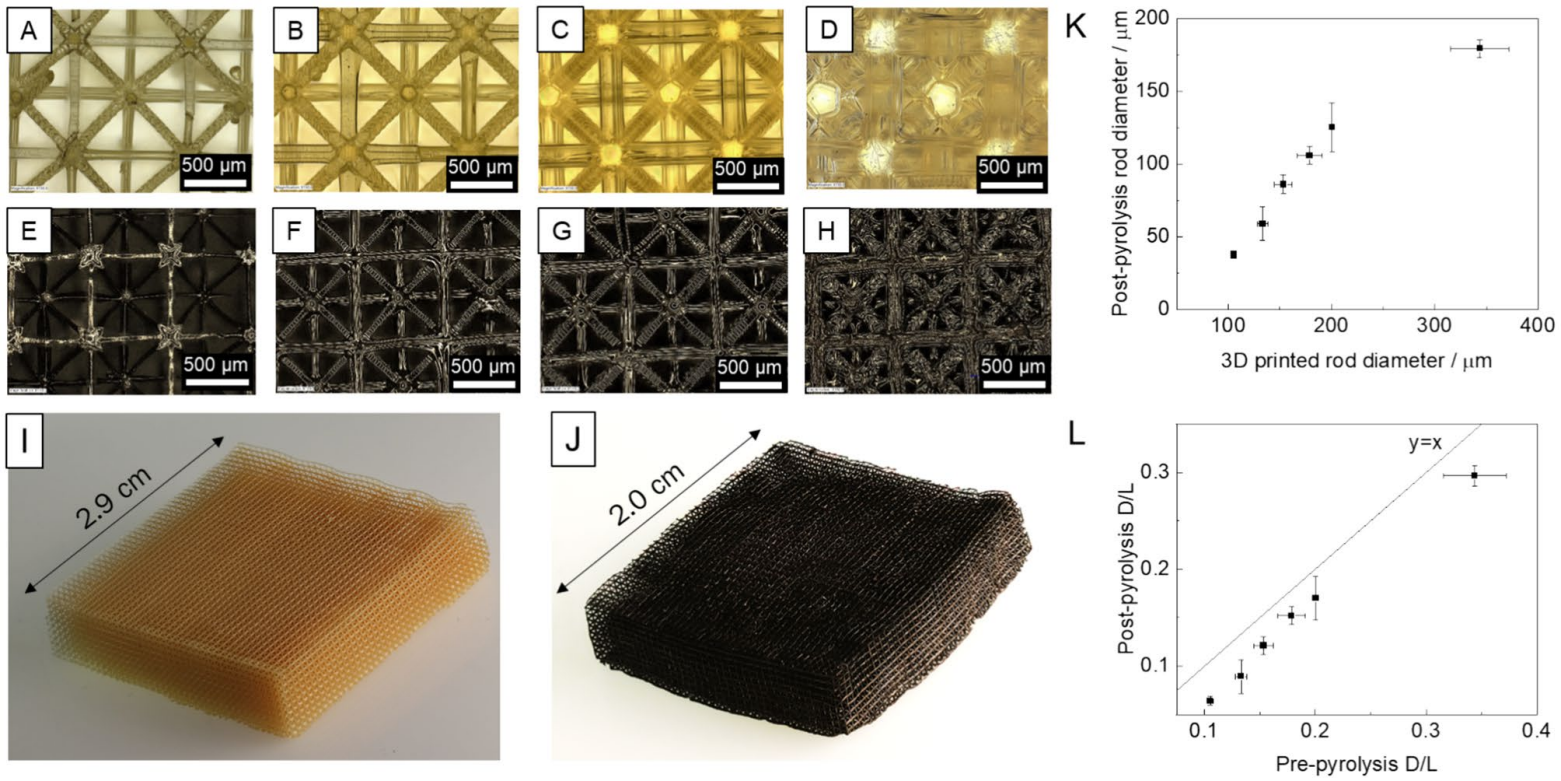
(**A–D**) Close up images of PuSL printed isotruss lattices for a target D/L ratio of 0.110, 0.140, 0.200, and 0.275, respectively. (**E–H**) Close up images of isotruss lattice electrodes after pyrolysis for a target D/L ratio of 0.110, 0.140, 0.200, and 0.275, respectively. (**I**) Oblique image of a lattice electrode with 29 × 29 × 7 unit cells (**J**) Image of lattice electrode after pyrolysis. (**K**) Measured beam diameter of 3D printed isotruss lattices as a function of target beam diameter. (**L**) Measured D/L after pyrolysis plotted as a function of D/L before pyrolysis.

Manufacturing an optimized electrode requires the ability to print a wide range of porosities with reproducible precision. We first demonstrate this by printing homogeneous lattices of isotruss unit cells which serve as the building block to control the porosity of the electrode. Herein, the term “homogeneous” refers to a lattice comprised of unit cells with a uniform specified rod radius throughout the entire network. Isotruss unit cells were chosen as the building block due to their mechanical strength and ability to be printed on PuSL printers^9,10,42,43^. The ratio of rod diameter to unit cell length, D/L, determines the local porosity and interfacial area of the 3D printed lattice. A printed unit cell length of 1 mm was chosen because of the wide range of possible beam diameters which could be printed on our PuSL system. These are shown in Fig. 3A–D.

After printing, the lattices undergo pyrolysis in order to reduce the printed polymer to non-graphitic carbon. This gives the lattices the electrical conductivity and catalytic activity necessary to function as a porous electrode. The shape and aspect ratio of the lattice was preserved throughout the pyrolysis process. Local images of the lattice after pyrolysis are shown in Fig. 3E–H. The pyrolysis process uniformly shrank the lattices by approximately 33%, so to account for this, printed lattices are scaled up accordingly such that the post-pyrolysis dimensions match the target dimensions. Features were preserved at the macroscopic structural scale, the unit cell scale, and the rod scale. Images of the macroscopic structure before and after pyrolysis are shown in Fig. 3I,J. At the unit cell scale, the shrinkage during pyrolysis was quantified using optical microscopy. Figure 3K shows the measured diameters of the rods before and after pyrolysis. The aspect ratio of rod diameter to unit cell length (D/L) is also preserved during the pyrolysis process. As shown in Fig. 3L, the D/L ratio after pyrolysis closely resembles the as-printed ratio, and scales linearly. Preserving this ratio is important, as D/L determines the local porosity and surface area of the electrode (Figure S1 in the Supporting Information). Measurement of the electrode conductivity after pyrolysis is provided in Figure S2 in the Supporting Information. Note that the focus of this work was to test electrodes that were as close to the simulated electrodes as possible. Of course, as seen in Fig. 3J, there are still some deformations. A more thorough sensitivity study of the effect of deformations due to pyrolysis on electrode performance would be worthwhile in the future, to examine, e.g., electrolyte flow that goes around the deformed electrode instead of through it and reduced contact with the current collector.

**Fig. 4:**
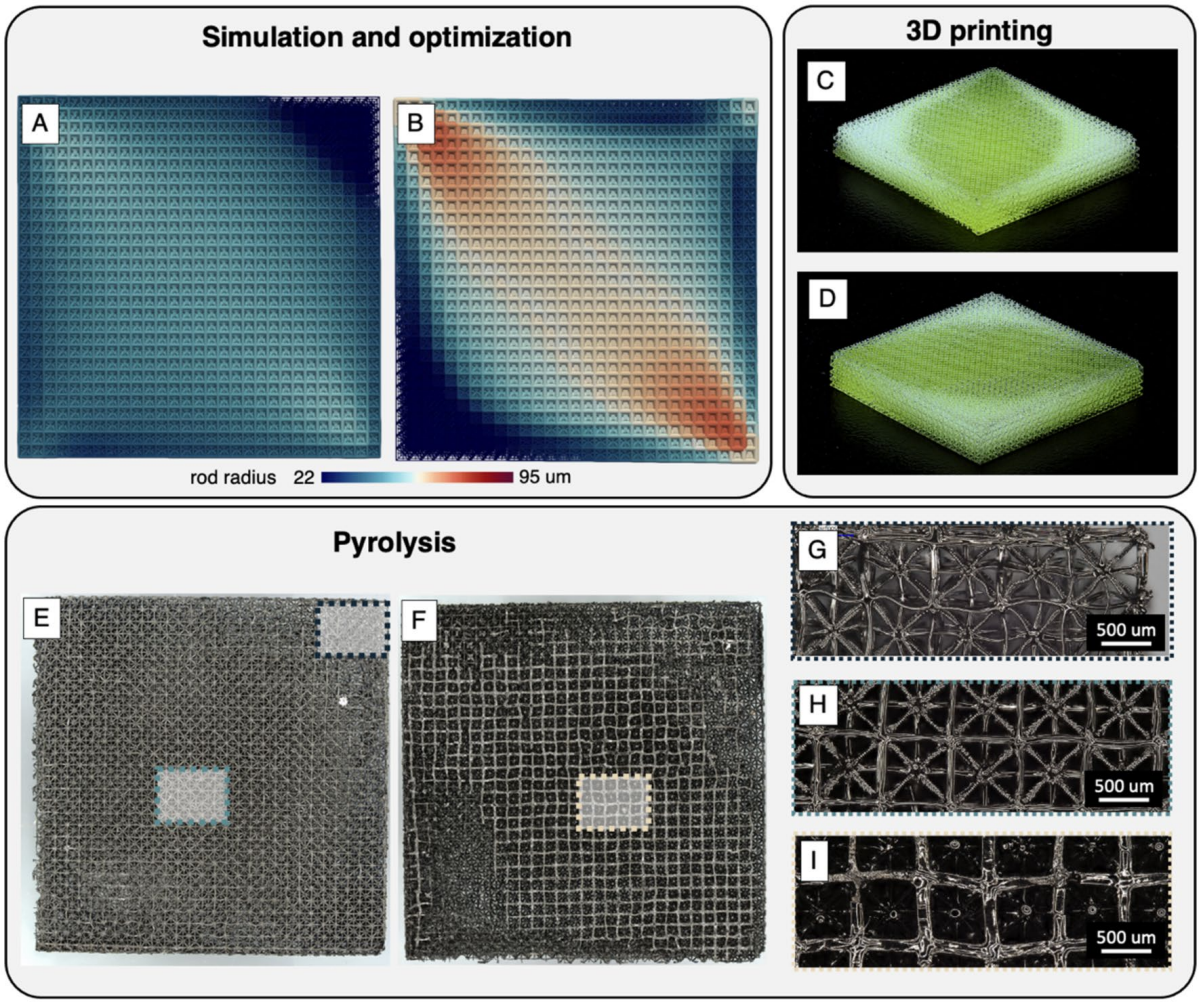
Structural confirmation of an electrode optimized for minimized power loss at a current density of 2.9 mA cm^−2^ and a flow rate of 50 mL min^−1^. (**A**) Top-side and (**B**) bottom-side schematic of beam radius distribution determined from simulations. (**C**) Oblique angle and (**D**) rotated views of 3D printed optimized electrode. (**E**) Top-side of the optimized electrode after pyrolysis. (**F**) Bottom-side of electrode after pyrolysis. Local images of the lattice structure were recorded for regions with (**G**) thin rods, (**H**) intermediate rods, and (**I**) thick rods.

Having verified the manufacture of homogeneous porosity electrodes, we now turn our attention to manufacturing a heterogeneous porosity electrode optimized for minimal power losses. A top and bottom view of the optimized electrode design is shown in Fig. 4A,B. The porosity shown is optimized for an electrolyte with a concentration of 1 mM ferricyanide flowing at 50 mL min^−1^ and an operating current density of 2.9 mA cm^−2^. A color map is used to highlight the trends in porosity across the structured electrode. At the inlet and outlet locations, the beams are thinnest, enhancing the electrode’s permeability to liquid flow. On the bottom side of the electrode, the beams are thickest, assisting with distributing the flow of reagents, ions, and electrons throughout the porous electrode. On the top side of the electrode, which rests against the membrane separator, the beams are intermediately sized, balancing the losses due to electron and ion transport with the active area needed to carry out the reaction.

Images of the electrode before and after pyrolysis verify that the electrode manufacturing process yielded the correct distribution in rod sizes. Images of the printed optimized electrode, prior to pyrolysis, are shown in Fig. 4C,D. The semi-transparent nature of the polymer causes light to absorb proportionately in response to changes in the local porosity. Therefore, the higher porosity regions near the inlet and outlet appear brighter than the denser regions in the middle and bottom side of the electrode. Macroscopic images of the optimized electrode after pyrolysis are shown in Fig. 4E,F. Insets of the local micro-structure, shown in Fig. 4G,H,I, are included to better highlight the spatial differences in porosity. Together, these images show that we can reliably design functional electrodes with a tunable local porosity tailored to meet the performance goals of the overall device. In the next section, we provide a more detailed look at how these changes in porosity result in measurable changes to the power requirements.

### Electrochemical validation

In the previous section, we demonstrated the ability to manufacture porous electrodes with spatially controlled porosities. In this section, we want to show how these changes in structure translate into performance improvements for the overall electrode. The model reaction for this comparison is the reduction of 1 mM potassium ferricyanide in 1 M KCl supporting electrolyte. The ferri and ferrocyanide redox couple is widely used as an electro-analytical redox system due to high reversibility and fast electrochemical response to carbon materials^44^. Three homogeneous electrodes were manufactured with different average rod radii (22, 66, and 102 μm) to probe the effect of average porosity. The homogeneous electrodes were then benchmarked against the performance of a heterogeneous porosity electrode which was computationally optimized for minimal total power loss when operating at a current density of 2.9 mA cm^−2^ and an electrolyte flow rate of 50 mL min^−1^.

**Fig. 5:**
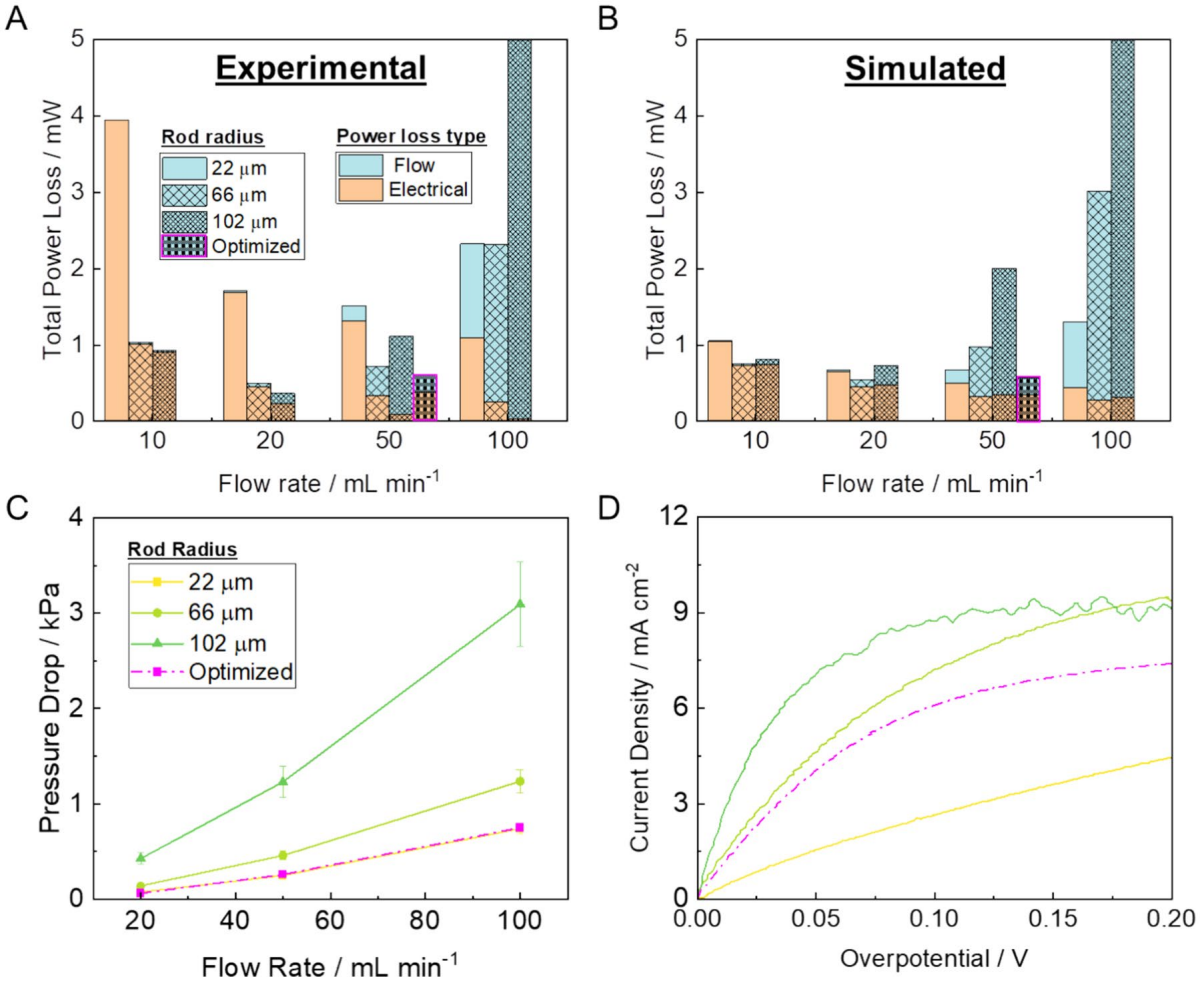
Performance of 3D printed flow through electrodes. (**A**) Experimental values of total power loss when the current density is 2.9 mA cm^−2^. (**B**) Simulated values of total power loss as a function of flow rate. (**C**) Measured pressure drop as a function of flow rate. (**D**) Polarization curves for lattice electrodes in electrolyte flowing at 50 mL min^−1^.

A summary of the experimentally observed total power losses incurred for the flow-through electrodes is shown in Fig. 5A. The clear changes in total power loss in response to both structure and electrolyte flow rate illustrate the mixed control apparent at these operating conditions. At lower flow rates, the electrical power losses dominate, partially due to the mass transport limitations and concentration gradients that develop throughout the porous electrodes. As the flow rate increases, these mass transport losses are alleviated, causing the electrical power losses to decrease. However, the pumping power required to maintain these electrolyte flow rates can be significant, and at 100 mL min^−1^, become the dominant form of power loss for the flow through electrodes.

A clear trend with structure also emerges when analyzing the total power loss. The thinnest rod radius electrodes are the most permeable to fluid flow, and therefore incur smaller flow power losses. Thicker radius electrodes have the most active surface area, causing them to generally incur the lowest electrical power losses. This highlights the fundamental tradeoff of porosity in flow through electrodes. The optimized electrode overcomes the limitations of this tradeoff by effectively allocating the rod distribution to balance the two power loss mechanisms. Highlighted in magenta in Fig. 5A, the optimized electrode exceeds the performance of the homogeneous porosity electrodes when operating at an electrolyte flow rate of 50 mL min^−1^. Compared to the performance of the 66 μm rod radius electrode, the optimized electrode is able to decrease the total power losses incurred by 16%.

These performance trends are also apparent in the simulations. Figure 5B shows the simulated total power loss of each electrode as a function of flow rate. Flowing electrolyte faster causes the electrical power losses to decrease while causing the flow power loss to increase. The magnitude of flow power loss tracks well with the experimentally reported values. There is a clear trend with electrode structure as well, with lower porosity electrodes incurring larger flow power losses. The order of magnitude for the electrical power losses was also comparable to what was observed experimentally. Refinements to the model, including microkinetic modeling which accounts for the surface scale morphology of the carbon rods, could help improve the fit between experiment and theory. Regardless of these subtleties, the simulation and experiment are aligned in predicting that the optimized electrode yields the lowest power losses for electrodes operating at the 50 mL min^−1^ flow rate it was designed for.

In practice, the optimized electrode can minimize power loss by mimicking the permeability of the thinnest radius electrodes, while harnessing the surface area and electrical activity of the thicker radius electrodes. For all electrode geometries, the pressure drop increases with both increasing flow rate and increasing rod radius. As shown in Fig. 5C, since many of the constituent beams of the optimized electrode are at or near 22 μm in radius, the pressure drop-flow rate relationship of the optimized electrode is nearly identical to that of the 22 μm lattice. This is advantageous given that the electrode was designed to operate at 50 mL min^−1^ flow rates, where the pressure drops can be significant. In addition to the improved permeability, the optimized electrode was also able to attain the electrical performance of the thicker radius electrodes. As shown in the polarization curves in Fig. 5D, the 102 μm rod radius electrode was able to draw the largest current densities in response to increasing applied overpotentials. By contrast, the 22 μm rod electrode required the largest overpotentials to reach the same current densities. The optimized electrode falls in between, most closely resembling the performance of the 66 μm electrode.

The tradeoff between flow power loss and electrical power loss shown in Fig. 5 underscores the complexity of designing and operating a flow reactor in mass transport limited regimes. It should be noted that the operating conditions—namely the reactant concentration, applied current density, and electrolyte flow rates—were chosen to create concentration gradients over the 2 cm × 2 cm × 0.5 cm dimensions of the printed flow through electrode. In a scaled-up reactor, these same concentration gradients and associated performance tradeoffs will persist. For example, state of the art redox flow batteries typically operate at current densities on the order of 100–1000 mA cm^−2^^45^, leading to higher electrical power losses. As the electrodes scale to larger footprints on the order of 1 m^2^, the pressure drop for fluid flow increases, leading to larger flow power losses^46,47^. Ultimately, the exact tradeoff between flow and electrical power loss at scale will depend on the specific redox chemistry and electrode geometry as well as other factors such as the integration of a flow field plate. To that end, 3D simulations and optimization studies can help with electrode design for systems at scale.

Overall, we have demonstrated that optimization can be an effective tool for designing manufacturable electrodes. Without the aid of optimization, we can certainly hypothesize using intuition that an electrode with some kind of mixture of low and high porosity regions can provide the benefits described above. However, there are far too many combinations of porosity distributions to test via trial-and-error in a reasonable time frame; this is further exacerbated when we want to eventually consider more industrially relevant scaled-up systems, since the design space only gets larger. Tools such as the ones presented in this manuscript can thus help future researchers develop electrode designs efficiently.

## Conclusions

In this manuscript, we have demonstrated the optimization, manufacturing, and electrochemical testing of a porous electrode. Specifically, we used computational optimization to design the porosity distribution of the electrode to minimize the flow and electrical power losses. To vary the porosity spatially within the electrode, we use the isotruss unit cell as the primary building block. This structure was then 3D printed using a PuSL system and pyrolyzed. The electrode was then electrochemically tested and compared to control electrodes of homogeneous porosity. We demonstrated that the optimized electrode reduced power losses by 16% at the operating current and flow rate it was specifically designed for.

This work demonstrates a new paradigm for designing porous electrodes. The proposed methodology accelerates reactor development by using computational tools to rapidly converge on an optimal pore structure. This optimal pore structure can be adapted to planned changes to the operating conditions, input parameters, and connections to external manifolding. The computationally generated structures were replicated in this study using 3D printing, but in principle structures could also be manufactured using more traditional and scalable methods. When designing reactors at scale, computational optimization can help determine the most important design motifs to prioritize. More complicated constraints or optimization frameworks could also be integrated to identify design considerations beyond sources of inefficiency. Such designs could account for machineability, corrosion resistance, or mechanical strength. For example, while Kudo et al.^34^ have shown similar pyrolyzed lattices with uniform beam radii, made with the same PR48 resin, to be strong in compression, the optimized lattices in this work that have not accounted for mechanics constraints may be less robust, e.g. due to regions of high porosity or warpage of the overall electrodes. The current work presented here can serve as a foundation for extensions such as these.

## Methods

### Experimental methods

#### Lattice design

Optimized porosity fields were generated using OpenFOAM simulations described below. Custom scripts were used to translate the porosity field into an LTCX file format, which describes the local porosity as a collection of isotruss unit cells with defined rod thickness. nTopology was used to mesh the LTCX files into an STL format, which can then be sliced into 3D-printable layers.

#### 3D printing

Commercial prototyping resin PR48 (Colorado Photopolymer Solutions) was used to print lattice electrode structures with a custom large-area projection microstereolithography printer. These printers, developed at Lawrence Livermore National Laboratory, can print fine feature sizes (approximately 25 µm) over large areas (approximately 12 cm × 12 cm × 4.5 cm) by scanning a focused image across the build plate to polymerize photosensitive resins layer by layer^9,10,48^. All lattices were printed with 25 µm layer heights. The printed lattices were washed by sonicating them for 5 min in isopropanol (Sigma-Aldrich). Afterwards, the lattices were heated to 190 ℃ for 4 h (Vulcan Benchtop Furnace, NeyTech) to thermally cure the printed polymer.

#### Annealing and carbonization

The printed PR48 electrodes were annealed in air at 300 ℃ for a few hours by ramping up from room temperature at 2 ℃ min^−1^ to undergo oxidative degeneration. During this process the acrylate polymer undergoes thermal decomposition and volatile products such as CO_2_, alcohol, and unsaturated hydrocarbons are released and initializes the cyclization of carbon back bone. Then, stepwise pyrolysis process is performed at temperatures of 250, 400, 600 and 1000 ℃ with a hold time of 1 h at each step and a heating ramp rate of 2 ℃ min^−1^ under N_2_ atmosphere. The annealed PR48 electrodes were ultimately converted to a form of pyrolytic carbon. During pyrolysis at 1000 ℃, between 500 and 700 ℃, a pyrolytic carbon is formed and at process temperatures above 700 ℃, C-C bonds are formed, and short-range ordering occurs^49^. If the pyrolysis process is not controlled the final structure will deform and collapse. Our method of combining oxidative degeneration followed by pyrolysis yields final parts with a volumetric shrinkage of 30%. The low temperature oxidative degeneration process is helpful in removing most of the volatile compounds prior to carbonization leading to minimal volume shrinkage.

#### Electrochemical methods

We have electrochemically tested these electrodes in a custom designed flow cell reactor using a mixture of 1 mM potassium ferri cyanide and 1 mM ferro cyanide dissolved in 1 M potassium chloride (Sigma Aldrich). The flow cell was designed using 2 graphite flow plates and Teflon end plates as shown in Figure S3 in the supporting information. An Ag/AgCl reference electrode (Pine Research) was placed near the working electrode through the graphite plate. To improve the hydrophilicity of the carbon lattice electrodes, they were treated using an air plasma cleaner (Harrick Plasma) for 30 s at a radio-frequency power of 18 W. Similar size of graphite felt (AVcarb) was used as the counter electrode. It was treated with 1 M sulfuric acid for 1 h, soaked in DI water overnight and rinsed with DI water. The working and counter electrode sides were separated using a cation exchange membrane (Nafion 211, Fuel Cell Store).

The electrolyte solution was degassed for 30 min using nitrogen and a blanket of nitrogen was provided throughout electrochemical testing to prevent the interference from dissolved oxygen. The electrolyte was circulated through the cell unit using a peristaltic pump (Cole Parmer) at a range of flow rates from 10 to 100 mL min^−1^.

We carried out 2 types of half-cell electrochemical studies, namely electrochemical impedance spectroscopy between working and reference electrodes at open circuit voltage (OCV) and linear sweep voltammogram from OCV to − 1 V vs Ag/AgCl at 1 mV/ s scan rates. Limited current densities were observed to compare performance of different electrodes at different flow rates.

The electrical power loss was calculated from experiments based on the product of the measured overpotential and current. At the beginning of an experiment, the open circuit potential (OCP) was measured between the working electrode and the reference electrode. The overpotential was defined as the voltage difference between the applied potential to the working electrode relative to OCP without IR correction.

#### Pressure drop experiments

The pressure drop through the electrode was measured by measuring the differential pressure between two pressure transducers (Omega) placed at the inlet and outlet of the electrode chamber. A syringe pump (Harvard Apparatus) was used to provide stable flow for the purposes of accurately quantifying pressure drop.

The flow power loss was calculated from experiments based on the product of the measured pressure drop and the flow rate. It should be noted that this flow power loss assumes a 100% pump efficiency, but in real systems the efficiency is likely to be in the range of 60–80%^50,51^.

### Computational methods

#### Fluid flow

The governing equations for the continuum homogenized electrochemical flow model are as follows. The fluid flow through the porous electrode is given by the Navier–Stokes equations with a Darcy drag,

$$\rho \varvec{u} \cdot \nabla \varvec{u} = - \frac{\mu }{\alpha \left( \gamma \right)}\varvec{u} - \nabla p + \mu \nabla^{2} \varvec{u},$$

$$\nabla \cdot {\varvec{u}}=0,$$

where ${\varvec{u}}$ is the fluid velocity and $p$ is the fluid pressure. In these equations, $\rho$ is the density, $\mu$ is the dynamic viscosity, and $\alpha \left(\gamma \right)$ is the local permeability, which depends on the design variable $\gamma$ used for optimization. The design variable is spatially dependent, $\gamma =\gamma \left({\varvec{x}}\right)$, where $\gamma =0$ corresponds to rods of radius ${r}_{min}$ and $\gamma =1$ corresponds to rods of radius ${r}_{max}$. At the inlet, we prescribe a velocity of ${\varvec{u}}=-\frac{Q}{{A}_{inlet}}{\varvec{n}}$, corresponding to a volume flow rate of $Q$, and the fluid exits to zero pressure $p=0$. All other boundaries in the simulation are no-slip boundaries where ${\varvec{u}}=0$**.**

The flow power loss from simulations was calculated by multiplying the bulk flow rate by the pressure difference across the inlet and outlet boundaries.

#### Mass transfer

The concentrations of the reductant and oxidant are given by advection–diffusion-reaction equations,

$${\varvec{u}}\cdot \nabla {C}_{i}=\nabla \cdot \left({D}_{i}\nabla {C}_{i}\right)+a\left(\gamma \right){j}_{n,i},$$

where species $i$ is either ${\left[Fe{\left(CN\right)}_{6}\right]}^{3-}$ or ${\left[Fe{\left(CN\right)}_{6}\right]}^{4-}$, ${D}_{i}$ is the effective diffusivity, $a\left(\gamma \right)$ is the available specific surface area available for reaction, and ${j}_{n,i}={k}_{m}({C}_{i}^{s}-{C}_{i})$ is the effective species flux from the solid to the bulk liquid. At all boundaries, the no flux condition is applied to the species concentration, except for the inlet, where the concentration is specified to be ${C}_{i}=1 \text{ mM}$.

#### Electrostatics

The solid and liquid potentials, ${\phi }_{1}$ and ${\phi }_{2}$, are modeled by the Poisson equations,

$$\nabla \cdot \left(-\sigma \nabla {\upphi }_{1}\right)=-a\left(\gamma \right){i}_{n},$$

$$\nabla \cdot \left(-\kappa \nabla {\upphi }_{2}\right)=a\left(\gamma \right){i}_{n},$$

where $\sigma$ and $\kappa$ are the effective solid and liquid conductivities. The exchange current density ${i}_{n}$ describing the redox reaction ${\left[Fe{\left(CN\right)}_{6}\right]}^{3-}+{e}^{-}\leftrightarrow {\left[Fe{\left(CN\right)}_{6}\right]}^{4-}$ with equilibrium potential ${U}_{0}$ is given by

$${i}_{n}=\frac{{i}_{0}}{{C}^{ref}}\left({C}_{{\left[Fe{\left(CN\right)}_{6}\right]}^{4-}}^{s}{e}^{\beta\Delta \phi }-{C}_{{\left[Fe{\left(CN\right)}_{6}\right]}^{3-}}^{s}{e}^{-\beta\Delta \phi }\right),$$

where $\beta =0.5F/RT$, where $F$ is Faraday’s constant, $R$ is the ideal gas constant, and $T$ is the temperature. The local overpotential is defined to be $\Delta \phi ={\phi }_{1}-{\phi }_{2}-{U}_{0}$. The exchange current density is related to the species flux ${j}_{n,i}$ via ${i}_{n}=F{j}_{n,{\left[Fe{\left(CN\right)}_{6}\right]}^{3-}}=-F{j}_{n,{\left[Fe{\left(CN\right)}_{6}\right]}^{4-}}$. At all boundaries, the no flux condition is applied to the potentials, except for the membrane surface, where a current $I$ over a membrane area $A$ is specified, $-\kappa \nabla {\phi }_{2}\cdot {\varvec{n}}=I/A$, and the current collector, where the potential of the solid is specified to be ${\phi }_{1}=0$.

The electrical power losses were calculated by multiplying the product of the total current and the overpotential measured at the back electrode boundary.

#### Homogenization

The porosity $\epsilon$, specific surface area $a$, permeability $\alpha$, and mass transfer coefficient ${k}_{m}$ have all been determined numerically by Beck et al. (2021), and we use the same values here. In Fig. S1 of the Supporting Information, the specific surface area and porosity are plotted against the nondimensional beam diameter. The Bruggeman correlation, shown in Figure S2 of the Supporting Information, is also used to approximate effective properties within the electrode,

$${D}_{i}={D}_{i,0}{\epsilon }^{3/2},$$

$$\kappa ={\kappa }_{o}{\epsilon }^{3/2},$$

$$\sigma ={\sigma }_{o}{\left(1-\epsilon \right)}^{3/2}.$$

## Supplementary Information

Supplementary Information.

## Data Availability

The datasets generated during and/or analyzed during the current study are available from the corresponding author on reasonable request.
