# Supplementary material for: 3D printed optimized electrodes for electrochemical flow reactors
Source: Sci Rep. 2024 Sep 30;14:22662. doi: 10.1038/s41598-024-71765-w (PMC11443137; doi:10.1038/s41598-024-71765-w)
Supplement: Supplementary file 1 — Supplementary Information. [file 41598_2024_71765_MOESM1_ESM.pdf]

Supporting Information

3D printed optimized electrodes for electrochemical flow reactors

Jonathan T. Davis, Buddhinie S. Jayathilake, Swetha Chandrasekaran, Jonathan Wong, Joshua R. Deotte, Sarah Baker, Victor Beck, Eric B. Duoss, Marcus Worsley, Tiras Y. Lin*

Lawrence Livermore National Laboratory, Livermore, California 94550, USA

*lin46@llnl.gov

Table of Contents

Contents

Porosity Correlations 2

Experimental Setup 3

Porosity Correlations

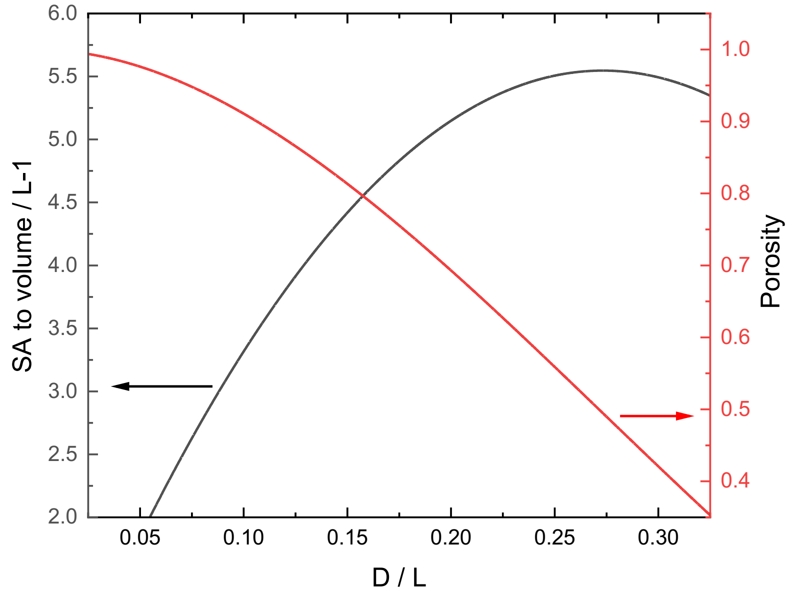

Figure S1. The specific surface area a and porosity ϵ plotted against the dimensionless beam diameter D/L.

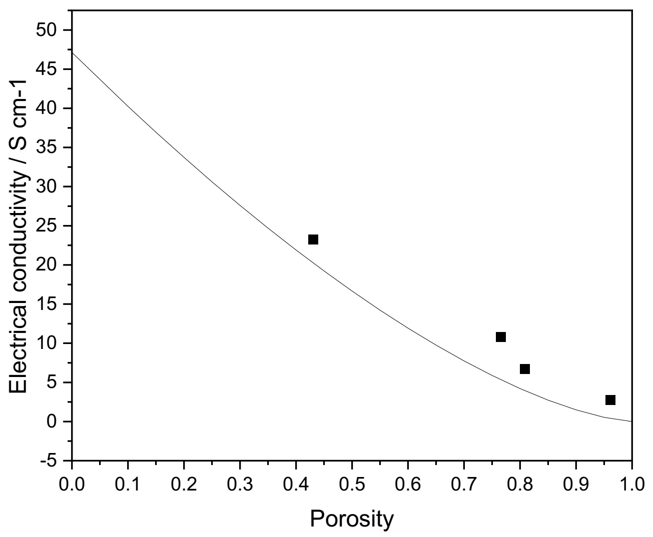

Figure S2. Correlation between electronic conductivity and electrode porosity. Individual points correspond to experimental data recorded using 4-point probe method. Solid line is fitted based on Bruggeman correlation.

Experimental Setup

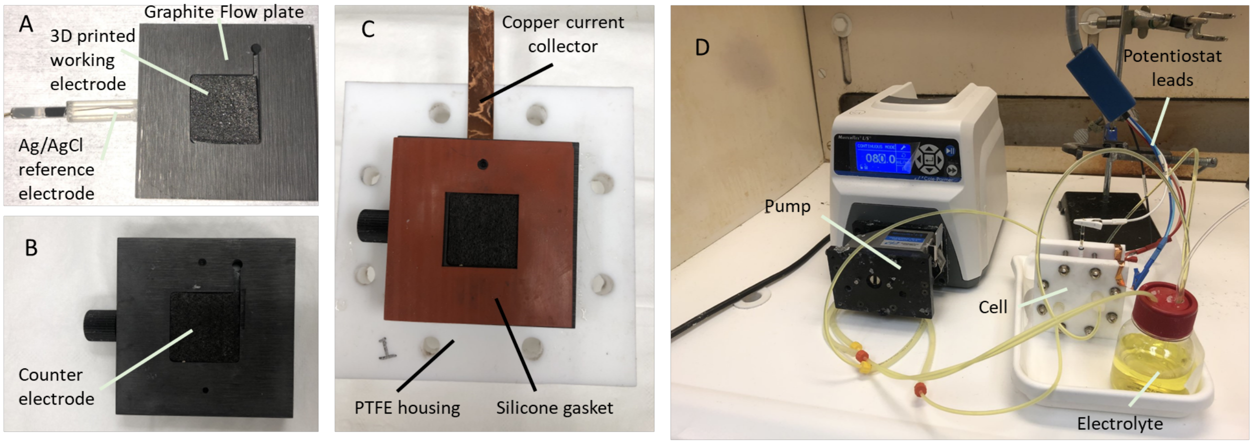

Figure S3: Experimental setup: (A) Working electrode plate (B) Counter electrode plate (C) Gasket-covered plate resting in PTFE housing. (D) Image of cell connected to electrolyte reservoir, peristaltic pump, and potentiostat leads.
